# Variation in Ringed Seal (*Pusa hispida*) Density Along a Latitudinal Gradient of Sea‐Ice Conditions

**DOI:** 10.1002/ece3.71472

**Published:** 2025-06-04

**Authors:** Cody G. Carlyle, James D. Roth, Brent G. Young, David J. Yurkowski, Christine Michel, Steven H. Ferguson

**Affiliations:** ^1^ Fisheries and Oceans Canada University Crescent Winnipeg Manitoba Canada; ^2^ Department of Biological Sciences University of Manitoba Winnipeg Manitoba Canada

**Keywords:** aerial survey, Arctic, climate change, last ice area, marine mammal, range

## Abstract

Anthropogenic climate warming is triggering poleward species redistributions, highlighting the importance of understanding how species distributions and abundance vary along latitudinal gradients. Ringed seals ( *Pusa hispida* ) rely on sea ice as habitat during key periods of their life history and inhabit a broad latitudinal range with diverse sea‐ice conditions, making them a model species to study patterns in density along a spatial–environmental gradient. We estimated the density of ringed seals from systematic aerial surveys along a latitudinal gradient in the eastern Canadian Arctic to investigate the response of ringed seals to regional variation in sea‐ice conditions. Ringed seals exhibited similar densities at our low (58.8° N; 2017: 0.46 ± 0.11 seals/km^2^) and intermediate latitude (72.7° N; 2016: 0.70 ± 0.14 seals/km^2^; 2017: 0.45 ± 0.07 seals/km^2^) regions. In contrast, observed ringed seal densities (2018: 0.05 ± 0.01 seals/km^2^; 2019: 0.09 ± 0.01 seals/km^2^) in the high‐latitude region (82.5° N) were an order of magnitude lower. This shift is concurrent with the transition in ice conditions from predominantly first‐year ice (85.0% concentration) at the low‐latitude region to primarily multiyear ice (86.8% concentration) at the high‐latitude region. These findings indicate that the variation in icescapes across the ringed seal's vast range likely has an influence on their density. We propose that ringed seal densities at higher latitudes are limited by multiyear ice, which is less suitable for construction of undersnow lairs and breathing holes. The shift in sea‐ice conditions may also have consequences for biological productivity that supports their diet. Our results suggest a nonuniform response of ringed seals to ongoing sea‐ice recession across the Arctic. Ringed seal densities could increase with a shift from multiyear to first‐year ice at higher latitudes and simultaneously decline with a transition from first‐year ice to open water at lower latitudes.

## Graphical abstract

Ringed seals ( *Pusa hispida* ) rely on sea ice for critical life‐history phases and inhabit a broad latitudinal range with diverse sea‐ice conditions, making them a model species to study patterns in density along a spatial‐environmental gradient using systematic aerial surveys across their range in the eastern Canadian Arctic. Ringed seals displayed an order of magnitude higher densities at lower latitudes compared to higher latitudes, concurrent with a transition from predominantly first‐year ice at lower latitudes to primarily multiyear ice at higher latitudes. These findings indicate that the variation in sea‐ice conditions across the ringed seal's range influences their density and highlights a likely non‐uniform response to ongoing sea‐ice recession across the Arctic, where their densities could increase with a shift from multiyear to first‐year ice at higher latitudes and simultaneously decline with a transition from first‐year ice to open water at lower latitudes.

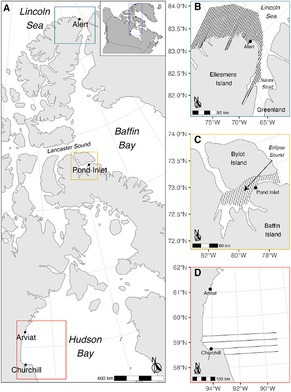

## 1 Introduction

Changing environmental conditions can have immediate beneficial or deleterious impacts on species distributions, but responses are not necessarily uniform in space and time (Laidre et al. 2020). Species distributions and range limits are strongly correlated with latitude, a proxy for abiotic factors such as light and temperature (MacArthur 1965; Stevens 1989). Anthropogenic climate change is altering these abiotic conditions, primarily temperature, to which species must respond by movement, tolerance, adaptation, or extinction. For example, climate warming is causing the poleward shifts of many species globally (Pecl et al. 2017). Understanding how species distributions, habitat use, and abundance vary over latitude is essential to predict how species respond to these environmental changes and detect alterations in ecosystem function (Frainer et al. 2017). Predicting the response of species and ecosystems is necessary to inform conservation efforts in the context of biological and socioeconomic impacts of continued environmental change (Pecl et al. 2017; Post et al. 2019).

The rate of warming in the Arctic is four times faster than the global average, and is causing a reduction in the total extent, thickness, and age of sea ice and fewer days of ice coverage (Jahn et al. 2024; Rantanen et al. 2022). Sea ice is fundamental to the Arctic marine ecosystem and its presence is strongly linked to latitude and temperature. Higher latitudes of the Arctic have higher concentrations of ice, more ice‐covered days, and thicker sea ice compared to lower latitudes. In addition, annual sea ice is present throughout the Arctic whereas older and thicker multiyear sea ice is typically found at higher latitudes, particularly the central Arctic Ocean. Multiyear sea ice continues to decrease rapidly, even in the “Last Ice Area” of northern Canada and Greenland projected to contain the last summer sea ice (Moore et al. 2019). Ice‐adapted species from sea‐ice algae to polar bears ( *Ursus maritimus* ) rely on specific sea‐ice conditions and phenology for energy acquisition and habitat (Post et al. 2013, 2019).

Ringed seals ( *Pusa hispida* ) experience large variation in sea‐ice conditions across their circumpolar range from the Subarctic to the central Arctic Ocean. Furthermore, ringed seals rely directly on sea ice as habitat for critical life‐history stages and are vulnerable to changes in sea‐ice conditions and timing (Kovacs et al. 2011; Laidre et al. 2008, 2015). Specifically, during the ice‐covered season, ringed seals prefer first‐year stable landfast or dense pack ice for the construction and maintenance of their subnivean (under snow) lairs and breathing holes for breeding, pupping, and nursing (Mclaren 1958; Smith and Hammill 1981), or can seasonally inhabit polynyas (Heide‐Jørgensen et al. 2013; Stirling 1997). Following the subnivean period, ringed seals still require stable landfast or consolidated first‐year ice for the energetically expensive annual molt (replacement of epidermal tissue and fur) (Mclaren 1958; Thometz et al. 2020). After the molt, ringed seals will spend the summer foraging intensively in open water (Young and Ferguson 2013) before setting up new territories for the subnivean period with sea‐ice freeze‐up in the fall. Years with more ice‐covered days and high ice concentrations have been associated with compromised breeding success and body condition in ringed seals in the western Canadian Arctic (Harwood et al. 2012). Conversely, years with low sea‐ice concentrations and fewer ice‐covered days correlate with decreased body condition and breeding success in Hudson Bay ringed seals (Ferguson et al. 2017), and potentially impact their molt by reducing preferred stable ice during this period (Thometz et al. 2020). Therefore, the variation in sea‐ice conditions experienced by ringed seals likely affects their regional densities and suggests that the response to climate change may not be uniform across their range (Ferguson et al. 2020; Laidre et al. 2020). In the well‐studied southern end of their range, ringed seals are expected to experience the impacts of sea‐ice recession via demographic shifts, diet changes, and range contraction (Ferguson et al. 2005, 2017; Young and Ferguson 2013). The more remote regions of the ringed seal's northern extent of their range are seldom studied due to the logistical challenges of conducting Arctic research (Laidre et al. 2015; Mallory et al. 2018). Overall, ringed seals are an interesting model to investigate differences in density over a large latitudinal and environmental gradient to better understand the impacts of climate change on Arctic species distributions.

Aerial surveys are an effective tool to study Arctic marine mammals over their vast distributions (Doniol‐ Valcroze et al. 2020). When systematically designed, aerial surveys are used to estimate abundance or densities of marine mammals using strip‐transect or distance sampling analysis and can be consistently repeated over time to monitor temporal changes in population abundance and distribution. For example, aerial surveys have commonly been used to estimate the relative abundance of ringed seals in the Canadian Arctic during the molting season when a higher proportion of ringed seals are basking on ice and available to count (Young et al. 2015). However, these estimates are considered relative indices of abundance due to uncertainties regarding the proportion of total seals observed during a survey. Several factors are associated with the proportion of seals on top of sea ice available to be observed, including survey date, time of day, weather (e.g., temperature, cloud cover, sun, wind speed, and direction), and predators (presence and avoidance strategies) (Bengtson et al. 2005; Carlens et al. 2006). Recent technological advancements have allowed aircraft to collect visible light and infrared images of the survey area to improve detection, eliminate visibility bias, and reduce costs and logistical constraints of observer‐based aerial surveys (Amstrup et al. 2004; Cameron et al. 2015). These digital survey methods have been applied successfully to improve the usefulness of aerial surveys as a tool to estimate relative abundance indices of ringed seals while hauled out over large areas throughout the Arctic (Conn et al. 2014; Young et al. 2019).

The aim of this study was to investigate the variation in the density of ringed seals across a latitudinal gradient from the northern to the southern extent of their range in the Canadian Arctic. We examined variation in ringed seal densities in relation to sea‐ice conditions across this latitudinal gradient using recently developed digital survey methods and sea‐ice data. We predicted that ringed seal densities would be higher in regions with greater availability of first‐year ice (landfast or consolidated), which is preferred for hauling out.

## 2 Methods

### 2.1 Survey Design

**FIGURE 1:**
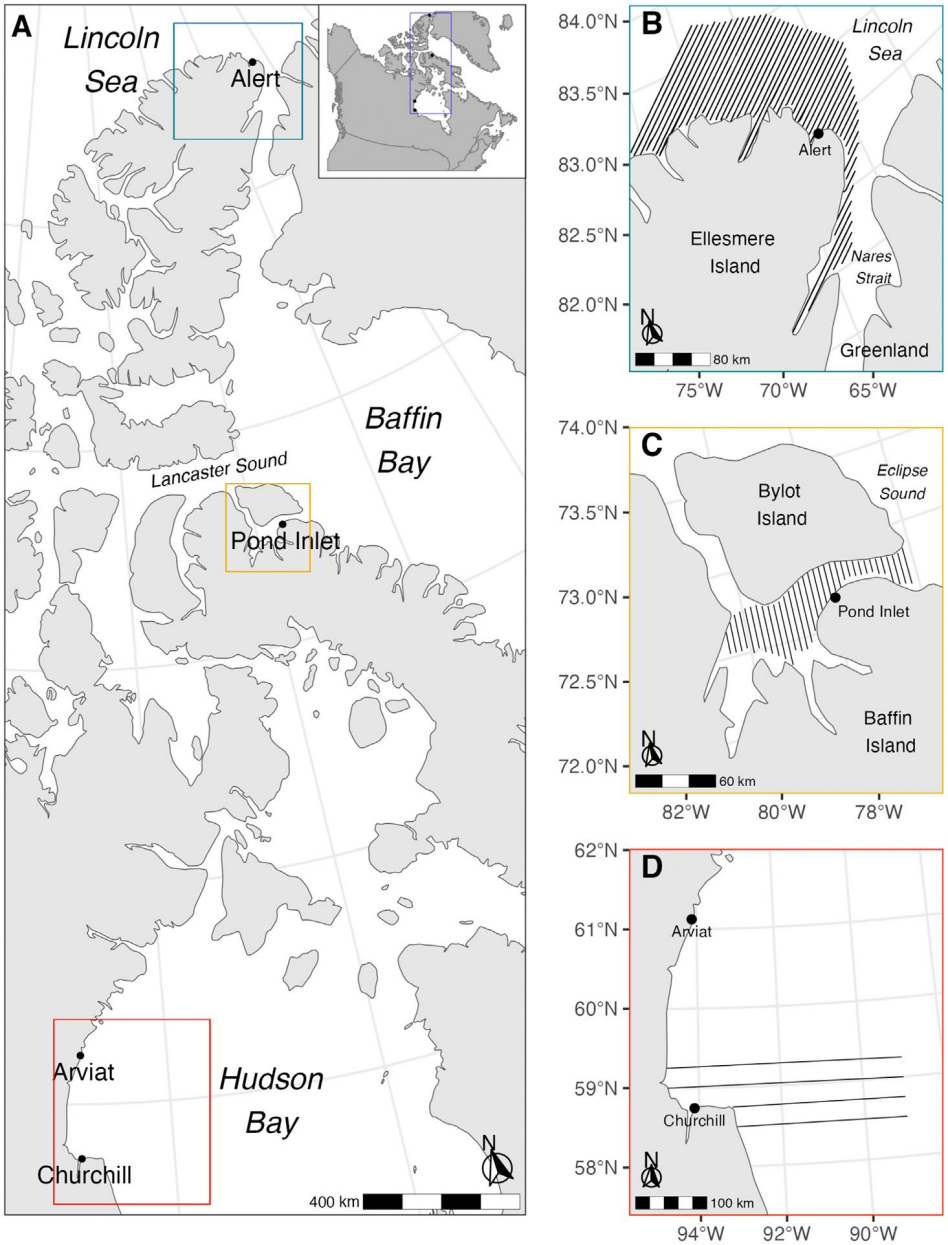
Aerial surveys of ringed seals hauled out on sea ice used to calculate relative density across a latitudinal gradient in the Canadian Arctic (A). Surveys were conducted near Churchill, Manitoba (58.8° N) to represent lower (D: Western Hudson Bay); Pond Inlet, Nunavut (72.7° N) to represent intermediate (C: Eclipse Sound/Lancaster Sound); and Alert, Nunavut (82.5° N) to represent higher latitudes of the Arctic (B: Lincoln Sea/Nares Strait). Solid lines represent transects that were successfully flown.

Aerial surveys were conducted at low (near Churchill, Manitoba, Canada: 58.8° N), intermediate (near Pond Inlet, Nunavut, Canada: 72.7° N), and high (near Alert, Nunavut, Canada: 82.5° N) latitude regions of the Arctic (Figure 1). Western Hudson Bay, near the southern end of the ringed seal's range, experiences seasonal coverage of entirely first‐year sea ice. The area is ice covered from December until break up begins in June with open water from July until freeze‐up begins in November (Gupta et al. 2022). At intermediate latitudes around Eclipse Sound and Lancaster Sound, first‐year ice is the dominant ice type, with intermittent presence of multiyear ice that is exported southward from the northern Canadian Arctic Archipelago (Howell et al. 2023). Sea‐ice freeze‐up begins in late September or early October for complete ice coverage in November until break up in late June or the beginning of July (Howell et al. 2024). Near the northern extent of the ringed seal's range, in the Lincoln Sea and Nares Strait region of the Last Ice Area, sea‐ice coverage is predominantly multiyear sea ice and is dense year‐round, although sea ice conditions in summer are very dynamic and shift between dense pack ice and open water on daily to weekly time scales (Moore et al. 2019). These three regions form a spatial and environmental gradient of low, intermediate, and high‐latitude regions of the Arctic and the ringed seal's range that covers a broad variation in sea‐ice conditions.

For each survey, strata were of an equal‐interval systematic strip‐transect design with a random start to create uniform coverage probability for robust estimation of relative density (Lunn et al. 1997; Young et al. 2015). The low‐latitude survey consisted of four parallel transects totaling 1134 km from the western Hudson Bay shoreline to 89°W longitude in the east and from slightly north to south of Churchill (Figure 1D; Table 1) (Lunn et al. 1997; Chambellant et al. 2012; Young et al. 2015). The intermediate‐latitude surveys consisted of 35 parallel transects totaling 874 km covering Eclipse Sound adjacent to Pond Inlet (Figure 1C; Table 1) (Young et al. 2019; Yurkowski, Young, et al. 2019). The high‐latitude surveys consisted of 51 parallel transects totaling 4074 km along the northern coast of Ellesmere Island surrounding Alert up to 131 km offshore in the Nares Strait and Lincoln Sea (Figure 1B; Table 1) (Yurkowski, Carlyle, et al. 2019; Carlyle et al. 2021). For the low‐latitude survey in 2017, weather allowed for completion of only four of the 10 transects typically used for density estimation (Figure 1D; Table 1) (Young et al. 2015). For the intermediate‐latitude region, every transect line was completed twice in 2016 and once in 2017 (Figure 1C; Table 1). For the high‐latitude location, we flew every transect line in 2018 and 2019 (Figure 1B; Table 1).

**TABLE 1:** Summary of digital‐based aerial surveys from our low‐, intermediate‐, and high‐latitude locations (2016–2019) (Carlyle et al. 2024; Young et al. 2019; Yurkowski, Young, et al. 2019) and past observer‐based aerial surveys for additional context (2007–2010, 2013) (Young et al. 2015).

Latitude (°N)	Year	Date	Method	Effort (km)	Transect spacing (km) (% cover)	Observations	# of seals	Density (SE)	CV	95% CI	Sea‐ice conc.	First‐year ice conc.	Multiyear ice conc.	Source
High (82.5)	2019	June 8–12	Digital	4073.8	5 (5)	100	116	0.09 (0.01)	12.6	0.07–0.12	98.3	7.2	87.2	This study
High (82.5)	2018	June 2–5	Digital	4067	5 (5)	44	49	0.05 (0.01)	17.6	0.03–0.07	98.8	13.2	86.8	This study
Intermediate (72.7)	2017	June 6–8	Digital	857.7	5 (5)	95	109	0.45 (0.07)	15.5	0.33–0.61	100	91.3	9.43	This study
Intermediate (72.7)	2016	June 17–22	Digital	1665.7	5 (5)	288 (June 17: 89, June 19/22: 199)	320 (June 17: 97, June 19/22: 223)	0.71 (0.05)	7.18	0.62–0.82	99.9	99.9	0.73	This study
Low (58.8)	2017	May 26–June 1	Digital	1133.5	27.8 (1.8)	150	191	0.46 (0.11)	23.6	0.29–0.72	85.5	85.0	0	This study
Low (58.8)	2013	May 28–June 5	Observer	3074.3	27.8 (1.8)	N/A	N/A	0.20	5.8	0.18–0.22	90.9	89.8	0	Young et al. 2015
Low (58.8)	2010	June 5–9	Observer	3074.3	27.8 (1.8)	N/A	N/A	0.73	10.5	0.59–0.89	64.6	64.4	0	Young et al. 2015
Low (58.8)	2009	June 2–8	Observer	546.4	27.8 (1.8)	N/A	N/A	0.28	34.2	0.14–0.53	63.7	63.7	0	Young et al. 2015
Low (58.8)	2008	May 28–31	Observer	2764.8	27.8 (1.8)	N/A	N/A	0.44	10.6	0.36–0.54	72.9	72.3	0	Young et al. 2015
Low (58.8)	2007	May 26–29	Observer	2869.5	27.8 (1.8)	N/A	N/A	0.92	8.0	0.78–1.07	90.3	90.2	0	Young et al. 2015

The surveys were conducted from late May to mid‐June (Table 1), during the molting season when an increased proportion of ringed seals have emerged from their lairs and are hauled out on the sea ice available to count (Mclaren 1958; Lindsay et al. 2021). Although the molting season usually occurs from mid‐May to mid‐July, the peak proportion of seals available for observation within the molting season likely occurs later in the season with increasing latitude to correspond with the timing of sea‐ice breakup and maximum solar insolation (Finley 1979) similar to pupping in Weddell seals (Stirling 1969). Local knowledge in Lunn et al. (1997) suggested that peak availability generally occurs in late May to early June for seals in our low‐latitude region, which they confirmed when surveys flown in mid‐June in western Hudson Bay were too late to observe seals on the sea ice. For seals in our intermediate‐latitude region, peak availability likely occurs closer to mid to late June (Finley 1979). Peak availability is unknown in our high‐latitude region but possibly occurs in late June or early July based on observations from previous aerial surveys and tagged seals (Kingsley et al. 1985; Born et al. 2002). Due to logistical constraints, our high‐latitude surveys were conducted in early to mid‐June (Table 1), possibly before the peak proportion of ringed seal availability. Information on ringed seal haul‐out proportions overlapping our study regions in space and time was not available to correct our survey estimates for variable availability. Within the constraints and potential limitations associated with aerial surveys, especially the timing of our high‐latitude surveys, we consider our estimates relative indices and not true densities. All surveys were flown between 0800 and 1800 local time and in low wind, cloud cover, fog, and precipitation, as adverse weather such as wind and cloud cover can cause variation in the proportion of time hauled out and the visibility of ringed seals (Bengtson et al. 2005). Density estimates can also be correlated with interannual variation in ringed seal movement into and out of our study areas (Finley 1979; Carlens et al. 2006). However, this effect is likely minimal as ringed seals display restricted home ranges and movement during the ice‐covered season (Born et al. 2004; Kelly, Badajos, et al. 2010; Luque et al. 2014; Yurkowski et al. 2016).

### 2.2 Digital Survey Protocol

We used digital survey methods to detect seals due to the improved detection and more simplified data collection, processing, and analysis of digital methods over traditional visual‐observer methods (Conn et al. 2014; Cameron et al. 2015; Young et al. 2019). Surveys were flown in a DeHavilland Twin Otter (DH‐6) aircraft at a target altitude of 305 m (1000 ft) and speed of 204 km/h (110 knots). A custom hole and mounting bracket in the belly of the aircraft was equipped with a Nikon (Tokyo, Japan) D810 digital single‐lens reflex (DSLR) camera with a 35 mm lens and a Forward Looking Infrared (FLIR; FLIR Systems Inc., Wilsonville, OR, USA) T1030sc camera with a 45°lens. The DSLR took an image every 2 s for an approximate 30% overlap between each successive image, and the FLIR took video at a rate of 5 frames every second. This continuous collection of visible light and infrared imagery of the area beneath the plane allowed a transect strip width of 312 m at an approximate ground sampling distance of 4.25 cm per pixel for the visible light photographs and a strip width of 250 m at an approximate ground sampling distance of 24.7 cm per pixel for infrared imagery. The 2019 high‐latitude survey used an array of two DSLR cameras instead of a DSLR and FLIR camera. A Bad Elf (West Hartford, CT, USA) global positioning system (GPS) was mounted in the plane and recorded the position, altitude, speed, and heading of the aircraft every second and connected to the Nikon through a Foolography (Berlin, Germany) Bluetooth unit, which allowed geotagging of every photo along the transect line. For each transect, the camera operator recorded the starting and ending image for the Nikon, the starting file and duration of each FLIR video, and a time stamp that linked each infrared image to each geotagged visible light image. A visual observer also documented environmental conditions such as ice concentration in tenths, cloud cover (%), fog, and precipitation.

### 2.3 Image Processing

Imagery was analyzed by a trained observer to locate seals hauled out on sea ice. Using FLIR Research IR Max software version 4.30.1.70 (FLIR Systems Inc., Wilsonville, OR, USA), the infrared video was scanned to detect heat signatures corresponding to observations of potential animals. Then the observer checked the GPS‐matched visible light image from the DSLR camera to validate the presence of ringed seal(s). Validation with images also allowed accurate recording of the number of seals associated with each observation. The high‐latitude survey in 2019 without FLIR video was analyzed for seals by scanning each individual DSLR image. This increases processing time but decreases seal detections by only about 3% (Young et al. 2019) when compared to processing with infrared video. GPS data associated with each image allowed a time and geographic position to be applied to all observations.

### 2.4 Density Estimation

Ringed seal detections on each transect were used in strip‐transect analysis of the 250 m strip below the plane covered by the FLIR camera. For the 2019 high‐latitude survey with only DSLR images, the 312 m strip captured by the DSLR camera was used. Strip‐transect analysis was performed according to Young et al. (2019) adapted from previously established methods for observer‐based aerial surveys (Chambellant et al. 2012; Young et al. 2015). Ringed seal density (seals/km^2^) was calculated using the standard ratio estimate according to Buckland et al. (2001).

$$\hat{D}=\sum_{i=l}^{k}n_{i}/\omega \sum_{i=l}^{k}l_{i}$$

In this equation, *k* is the number of transects flown, *n*_*i*_ is the number of ringed seals on the *i*th transect, *ω* is the strip width, and *l*_*i*_ is the length of the *i*th transect. The length of the transects was calculated using the GPS locations attached to images collected to ensure density was calculated based on the realized flown survey effort. The variance, coefficient of variation (CV), and 95% confidence intervals of the density were also calculated according to Young et al. (2019) adapted from methods developed for observer‐based aerial surveys (Kingsley and Smith 1981; Buckland et al. 2001; Chambellant et al. 2012). We also compared our findings with past results from observer‐based aerial surveys in our low‐latitude region (2007–2010, 2013) (Table 1) (Lunn et al. 1997; Chambellant et al. 2012; Young et al. 2015) to provide context to our limited digital survey estimates.

### 2.5 Sea‐Ice Concentration

We accessed the Canadian Ice Service weekly regional ice data product (https://iceweb1.cis.ec.gc.ca) that corresponded most closely with each day of the aerial surveys. Using the geotagged survey coverage and the known strip width captured by the survey cameras, we extracted the sea‐ice concentration and ice type for the covered survey area using R version 3.4.2 and QGIS version 3.12.2 (R Core Team 2020). We then calculated the mean concentration (%) of total sea ice, first‐year ice, and multiyear ice for the covered area of each survey.

### 2.6 Statistical Analysis

We used a generalized linear model (GLM) with a Gamma distribution and an inverse link function for zero‐truncated continuous data to investigate spatial differences in ringed seal density, which was compared among regions as a categorical variable using Tukey post hoc analysis. To explore the role that our limited sample size (*n* = 5 surveys) may have had in a lack of significance in the GLM performed, we conducted post hoc power analyses for our desired *α* and a suggested power (1‐β) of 0.8 (Cohen 2013). We used two separate simple linear regressions to investigate the relationship between the variation in seal density and the mean (1) first‐year and (2) multiyear ice concentration calculated for each survey. Total sea ice concentration was removed from analysis due to low variation between regions (the range for 4 of 5 surveys was 98.2%–100% and all surveys had a range of 85.5%–100%) (Table 1). Results for all tests were considered significant at *α* = 0.05. Models were validated and model assumptions were tested by examining the residuals against the fitted values for covariates of interest to ensure valid model specification (e.g., location, survey date, proportional effort) (Zuur and Ieno 2016). All statistical analyses and data visualization were performed using R version 3.4.2 and packages “ggplot2,” “ggpubr,” and the “tidyverse,” except for the power analysis, which was performed using GPower 3.1 (Faul et al. 2007; Erdfelder et al. 2009; Kassambara 2020; R Core Team 2020; Wickham 2016; Wickham et al. 2019).

## 3 Results

**FIGURE 2:**
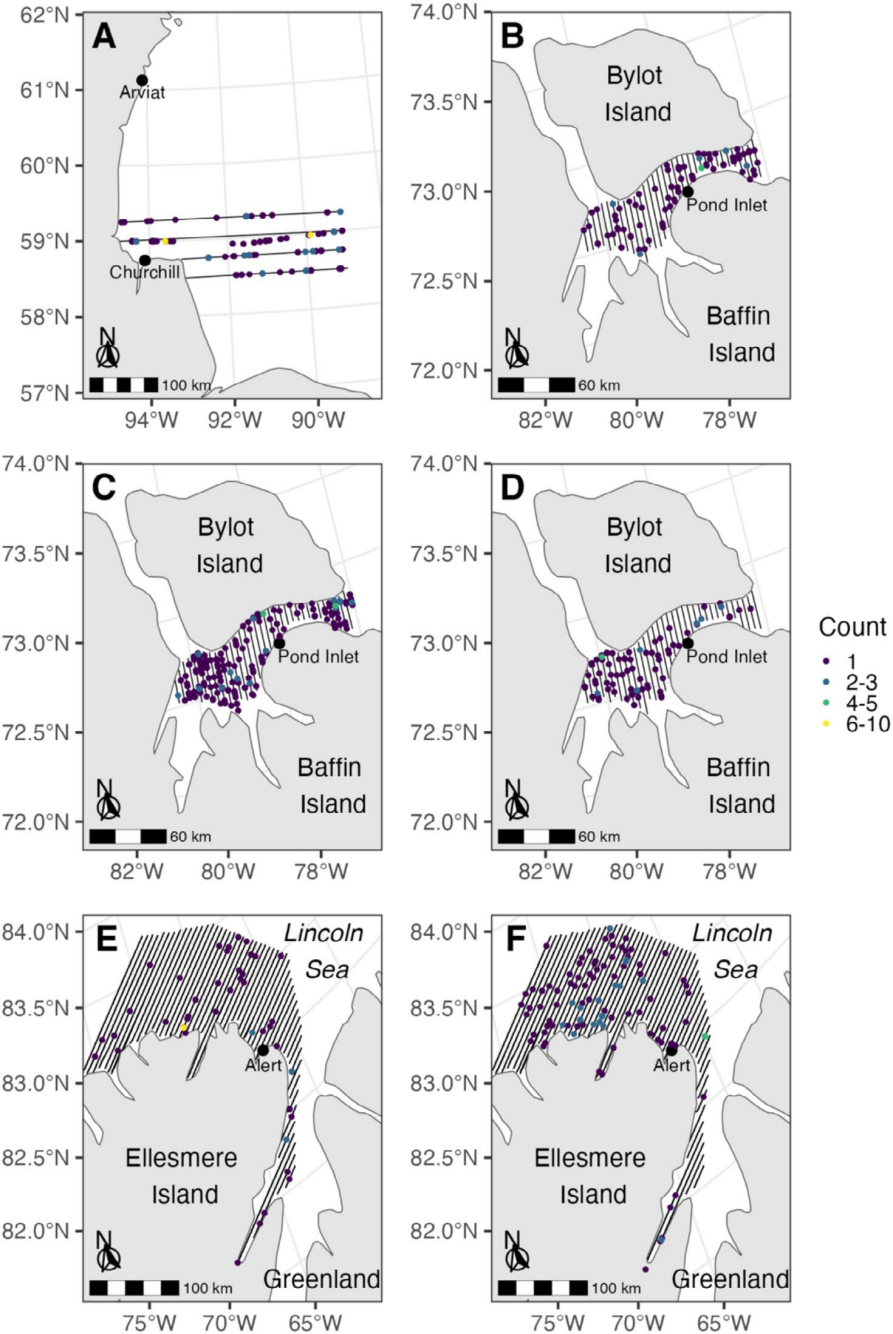
Ringed seal observations recorded by infrared and visible light photography from digital‐based aerial surveys in low (2017: A), intermediate (2–5 June 2016: B; 17–22 June, 2016: C; 2017: D) and high (2018: E; 2019: F) latitude regions.

During the low‐latitude survey in 2017, 191 ringed seals were counted from 150 observations (Figure 2; Table 1). In the 2016 intermediate‐latitude survey, there were a total of 320 seals (June 17: 97, June 19/22: 223) from 288 observations (June 17: 89, June 19/22: 199) during the two completions of the transect lines (Figure 2; Table 1). We counted 109 ringed seals in 95 observations during the 2017 survey in the intermediate‐latitude location (Figure 2; Table 1). In the high‐latitude location, we counted 49 ringed seals from 44 observations in 2018 and 116 ringed seals from 100 observations during 2019 (Figure 2; Table 1).

**FIGURE 3:**
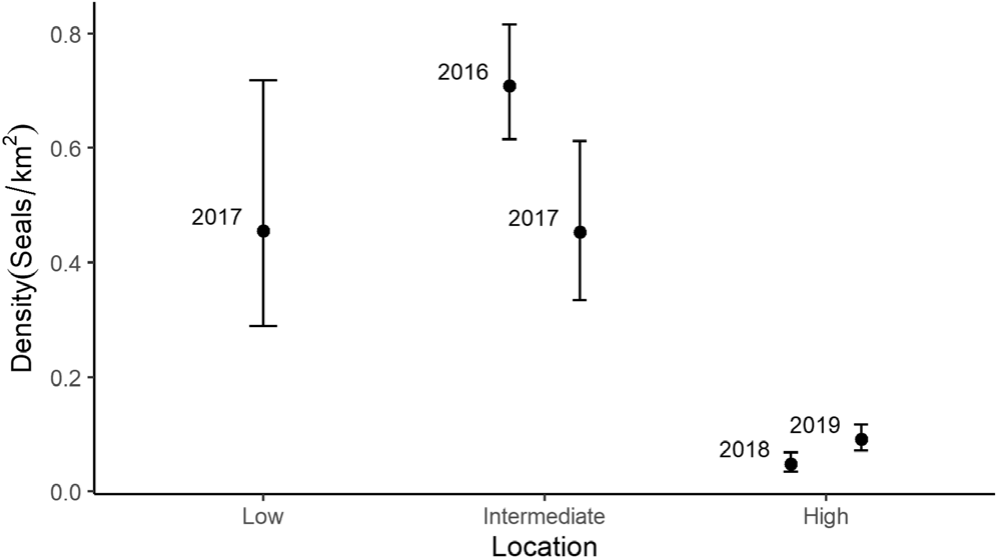
Ringed seal density estimates (means and 95% confidence intervals) calculated by strip‐transect analysis from digital‐based aerial surveys in low (2017), intermediate (2016–2017), and high (2018–2019) latitude regions.

**FIGURE 4:**
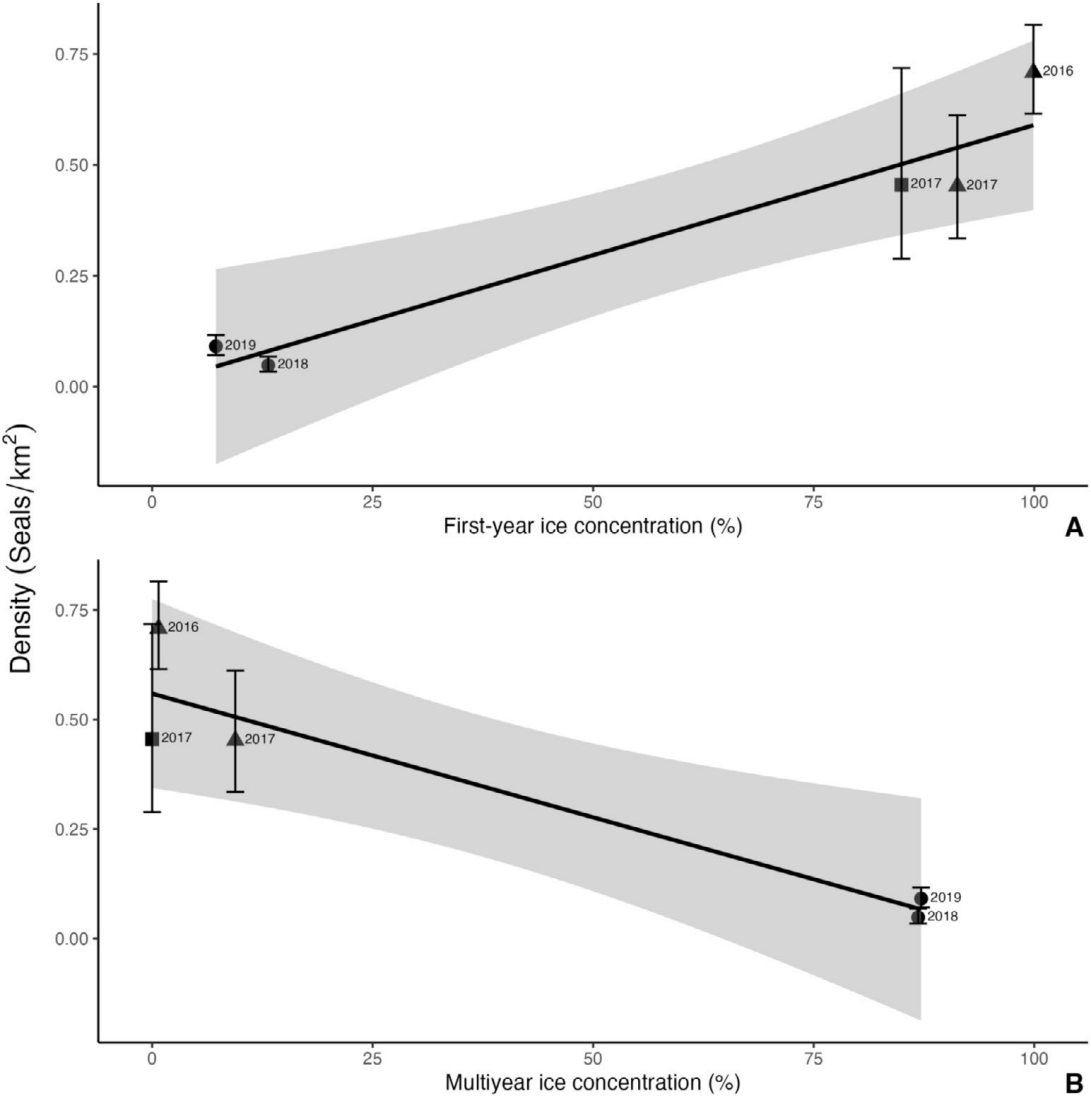
Ringed seal density estimates (means and 95% confidence intervals) related to (A) first‐year ice concentration (*β* = 0.0059, *F*_1,3_ = 31.37, *p* = 0.011, *R* ^2^ = 0.91), and (B) multiyear ice concentration (*β* = −0.0057, *F*_1,3_ = 21.2, *p* = 0.019, *R* ^2^ = 0.88). Digital‐based aerial surveys were conducted in low (squares), intermediate (triangles), and high‐latitude (circles) regions.

Strip‐transect analysis indicated seal densities in the high‐latitude location (2018: 0.05 ± 0.01 seals/km^2^, 2019: 0.09 ± 0.01 seals/km^2^) were approximately an order of magnitude lower than in the intermediate (2016: 0.70 ± 0.14 seals/km^2^, 2017: 0.45 ± 0.07 seals/km^2^) and low (2017: 0.46 ± 0.11 seals/km^2^) latitude locations, while the interannual variability ranged between 1.6–1.8× in density within locations (Figure 3; Table 1). Moreover, a Tukey post hoc test indicated that ringed seal densities in the high‐latitude location were lower than in the intermediate (*z* = 3.262, *p* < 0.005) and low (*z* = 3.091, *p* < 0.005) latitude locations, whereas densities in the low and intermediate‐latitude locations were not different from each other (*z* = −0.498, *p* = 0.861). Although location was not a statistically significant predictor of density as determined by the full GLM (F_2,4_ = 13.7, *p* = 0.068), a post hoc power analysis indicated that, based on the effect size observed in our study, only an additional two samples (*n* = 7) would have been required to achieve statistical significance. Ringed seal density significantly increased with first‐year ice concentration (*R* ^2^ = 0.91, *F*_1,3_ = 31.37, *p* = 0.011) and significantly decreased with multiyear ice concentration (*R* ^2^ = 0.88, *F*_1,3_ = 21.2, *p* = 0.019) (Figure 4).

## 4 Discussion

This study is the first to investigate spatial variation in ringed seal density across nearly the entire northern to southern extent of their range (approximately 59°N–83°N) and in relation to varying regional sea‐ice conditions using multiple annual aerial surveys. Our extensive coverage of ringed seal density over their entire latitudinal range validates smaller scale observations that the type of ice matters in addition to the amount of sea ice (Kingsley et al. 1985). Specifically, the lower ringed seal density observed in our high‐latitude region parallels the limited availability of preferred first‐year ice and the dominance of multiyear ice in the region. In contrast, the higher ringed seal densities in our low and intermediate‐latitude regions match a high availability of first‐year ice and a relative absence of multiyear ice. This result supports the hypothesis that the densities of ringed seals across their latitudinal range in the Arctic would reflect access to preferred first‐year sea‐ice habitat (Kingsley et al. 1985; Smith and Hammill 1981).

First‐year ice or thinner ice habitat is preferred by ringed seals for building and maintaining subnivean lairs and breathing holes by scraping holes in the ice with their claws, and for the formation of small holes and cracks used by ringed seals to facilitate these structures (Hammill and Smith 1989; Smith and Hammill 1981). Primarily multiyear ice and sparse first‐year ice habitat could limit their density at higher latitudes (Kovacs et al. 2011). Our goal was to compare broader differences between regions, so we did not analyze within‐region patterns, though we did observe support for the preference of first‐year ice over multiyear ice by ringed seals in our high‐latitude region (Figure S1). At lower latitudes, greater open water and limited availability of stable first‐year ice could directly impact the success of pupping and nursing (Ferguson et al. 2005; Iacozza and Ferguson 2014; Smith and Harwood 2001), and extend the energetically costly molting period (Thometz et al. 2020). A favorable zone where environmental characteristics best match ringed seal habitat requirements (Ferguson et al. 2020) may exist between these two thresholds in sea‐ice conditions. Lower densities of ringed seals in our high‐latitude region may also be related to lower algal productivity. Increased sunlight in spring coincident with sea ice melt results in a burst of algal productivity in Arctic ecosystems (Post et al. 2013; Tedesco et al. 2019). Ringed seals rely on the seasonal pulse in prey driven by this sea‐ice algal bloom to rebuild energy stores (Carlyle et al. 2022; Koch et al. 2023) after the energetically expensive molt (Young and Ferguson 2013; Thometz et al. 2020). The dominance of multiyear ice and more ice‐covered days at higher latitudes of the Arctic is linked to the magnitude and timing of algal productivity (Tedesco et al. 2019; Campbell et al. 2022), which could reduce prey available to ringed seals (Harwood et al. 2012). In addition to sea‐ice conditions, other factors that covary with latitude such as solar radiation can impact the productivity of Arctic marine ecosystems (Tedesco et al. 2019). These other factors also likely contributed to a less suitable environment for ringed seals and limited the seal densities we observed at higher latitudes.

Our ringed seal density estimates from digital surveys in the high‐latitude region are also lower than density estimates from all previous surveys in the low‐latitude region (Young et al. 2015) (Table 1). This strengthens our conclusion that ringed seal densities were lower at the highest latitude region. (Young et al. 2019). This comparison with past surveys also highlights that a potential decline in ringed seal densities in the low‐latitude region since 1995 proposed by Young et al. (2015) and Ferguson et al. (2017) may be continuing. The observer‐based density estimate for the low‐latitude region in 2017 was 0.2 seals/km^2^ (Young et al. 2019), which continues the declining trend when compared to the observer‐based estimates from Young et al. (2015) and Ferguson et al. (2017). A decline in ringed seal densities at the low‐latitude region would parallel recent observed demographic and diet shifts in ringed seals in the region (Ferguson et al. 2017) and may reflect aforementioned impacts of an increase in open water relative to first‐year ice (Gupta et al. 2022) beyond a favorable zone in western Hudson Bay.

Many factors could have affected the number of seals we detected in each region during our aerial surveys. Survey date, time of day, location, weather (e.g., wind speed), presence of predators, and ice conditions impact the proportion of seals available for observation and introduce negative bias to the density estimates (Bengtson et al. 2005; Carlens et al. 2006; Kelly et al. 2006; Lindsay et al. 2021). For this reason, our estimates are relative indices rather than absolute densities. With an understanding of these limitations, standardized surveys are still useful to understand ringed seal densities and distributions during the spring ice‐covered season and early breakup, which can be replicated over time to document the response of ringed seals to continued environmental and ecological change (Young et al. 2015). The 2018 (2–5 June) and 2019 (8–12 June) surveys in the high‐latitude region were too early to capture the peak proportion of seals available for observation and likely underestimate seal densities, but not to the same extent as before and after snow‐lair emergence (Kingsley et al. 1985; Kelly et al. 2006; Lindsay et al. 2021). Up to five to seven‐fold increases in visible seals have been reported when comparing ringed seal availability before and after snow‐lair emergence occurs in April or May in Alaska (Kelly et al. 2006; Lindsay et al. 2021). However, seals were observed on top of ice and along cracks during both high Arctic surveys and as early as 8 May in 2019 (11 month before surveys) indicating our high‐latitude surveys occurred after lair emergence when ringed seals spend more time hauled out for molting (Born et al. 2002). This postemergence haul‐out period can last for several weeks to over a month to coincide with increased solar radiation and has relatively constant proportions of seals available for observation (Finley 1979). Therefore, it is unlikely that the negative bias of our high Arctic survey estimates approaches the magnitude needed for the several‐fold (5‐14×) increase in ringed seal density to be statistically similar to the intermediate and low‐latitude regions. Future surveys conducted throughout the molting season could improve our understanding of seasonal changes in seal availability.

Although outside the focus of our regional comparison, interannual variation in seal densities within our regions was also observed (Figure 3; Table 1). Since ringed seal life history is characterized by high adult survival resulting in relatively long life for their body size (Ferguson et al. 2024), rather than population change, interannual differences in density are likely due to changes in availability bias or movement into and out of our study areas (Bengtson et al. 2005; Carlens et al. 2006). These within‐region patterns of availability or movement could be associated with variation in sea‐ice conditions at local scales. In 2018, survey observers in our high‐latitude region noted sea‐ice concentrations of 80%–100% with sparse leads throughout the survey, while in 2019 observers noted a mix of areas with 70%–100% sea‐ice concentration and areas with open water (0%–10%) or drift ice (10%–60%). These observations coincided with a notably earlier breakup in ice bridges in the Nares Strait and Smith Sound in 2019 (Vincent 2019). These ice bridges block sea ice (especially thicker and older ice) from being exported out of our study site through the Nares Strait (Vincent 2019). Therefore, early breakup allows for increased sea‐ice export, impacting the sea‐ice conditions in the region. We propose that in addition to the later survey dates, the difference in sea‐ice conditions likely increased the proportion of seals available or movement of seals (Finley 1979) into the surveyed area in 2019 compared to 2018, resulting in higher observed ringed seal densities. Future research focused on understanding interannual variation in ringed seal density and behavior in these dynamic sea‐ice environments (Moore et al. 2019) could quantify the distribution of ringed seals in relation to sea‐ice variables (concentration, ice type, flow velocity) at finer spatial and temporal resolution.

In conclusion, our study informs the crucial relationship between ringed seals and the highly variable sea‐ice environments they rely on for key life‐history stages (Laidre et al. 2008). Ringed seal densities varied with spatial variations in their habitat, highlighting the need for caution in predicting species responses to environmental change over their entire range. Our findings suggest that changes in ringed seal density may not follow a uniform trend across their vast range. Further monitoring is needed to validate our conclusions before robust predictions can be made about the response of ringed seals to sea‐ice recession. At higher latitudes where they currently experience high concentrations of multiyear ice and a long ice‐covered season, uncertainties persist regarding how these seals will adapt to the complete replacement of multiyear ice by first‐year ice projected by mid‐century (Ferguson et al. 2024; Jahn et al. 2024) and associated increase in algal productivity (Arrigo and van Dijken 2015; Tedesco et al. 2019). This uncertainty is compounded by potential impacts of sea‐ice loss on Arctic food webs and the availability of essential prey such as Arctic cod (Carlyle et al. 2022; Koch et al. 2023), and emphasizes the potential importance of the Last Ice Area as a refuge for ice‐adapted species (Laidre et al. 2020; Moore et al. 2019). At lower latitudes, where sea‐ice loss threatens to replace preferred first‐year ice with open water and earlier spring breakup, observed declines in ringed seal densities may persist (Young et al. 2015, this study). Indeed, the threat of sea ice loss to breeding and molting habitat of ringed seals has led to circumpolar countries recommending the ringed seal be listed as “special concern” (Kelly, Bengston, et al. 2010; COSEWIC 2019). The intricate interdependence between ringed seals, polar bears (Stirling and Archibald 1977), and Arctic communities (Wenzel et al. 2016) underscores the urgency of understanding and mitigating the impacts of ongoing environmental changes on these vital Arctic ecosystems.

## Author Contributions

**Cody G. Carlyle:** conceptualization (equal), data curation (equal), formal analysis (lead), investigation (lead), methodology (equal), validation (lead), visualization (lead), writing – original draft (lead), writing – review and editing (lead). **James D. Roth:** formal analysis (supporting), investigation (supporting), supervision (equal), validation (supporting), writing – original draft (supporting), writing – review and editing (supporting). **Brent G. Young:** data curation (equal), formal analysis (supporting), methodology (equal), validation (supporting), writing – original draft (supporting), writing – review and editing (supporting). **David J. Yurkowski:** conceptualization (equal), formal analysis (supporting), investigation (supporting), validation (supporting), writing – original draft (supporting), writing – review and editing (supporting). **Christine Michel:** funding acquisition (equal), project administration (equal), resources (equal), writing – original draft (supporting), writing – review and editing (supporting). **Steven H. Ferguson:** conceptualization (equal), data curation (equal), formal analysis (supporting), funding acquisition (equal), investigation (supporting), methodology (equal), project administration (equal), resources (equal), supervision (equal), validation (supporting), writing – original draft (supporting), writing – review and editing (supporting).

## Conflicts of Interest

The authors declare no conflicts of interest.

## Supporting information


Figure S1.

## Data Availability

The data that support the findings of this study are presented in part (i.e., whole survey densities, sea‐ice concentrations) in Table 1 and openly available in its entirety on Dryad at https://doi.org/10.5061/dryad.7h44j103j. Sea‐ice concentration data were derived from the Canadian Ice Service weekly regional ice data product available in the public domain [https://iceweb1.cis.ec.gc.ca].
