# Supplementary material for: Variation in Ringed Seal (Pusa hispida) Density Along a Latitudinal Gradient of Sea‐Ice Conditions
Source: Ecol Evol. 2025 Jun 4;15(6):e71472. doi: 10.1002/ece3.71472 (PMC12137619; doi:10.1002/ece3.71472)
Supplement: Supplementary file 1 — Figure S1. [file ECE3-15-e71472-s001.docx]

**
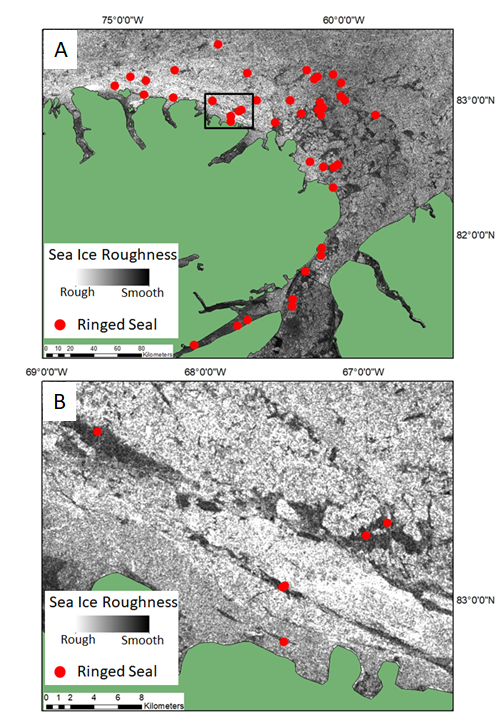
**

**Figure S1.** (A) Ringed seal observations (red points) from the high Arctic aerial survey carried out from 2-5 June, 2018 and (B) a close up of five ringed seal observations on 5 June, 2018 overlaid with Radarsat-2 imagery. Lighter values indicate rougher surface (i.e., likely multiyear ice) and darker values indicate smoother surface (i.e., likely first-year ice or cracks)
